# The development of dasatinib as a treatment for chronic myeloid leukemia (CML): from initial studies to application in newly diagnosed patients

**DOI:** 10.1007/s00432-013-1488-z

**Published:** 2013-08-13

**Authors:** Andreas Hochhaus, Hagop Kantarjian

**Affiliations:** 1 Abteilung Hämatologie/Onkologie, Klinik für Innere Medizin II, Universitätsklinikum Jena, Erlanger Allee 101, 07740 Jena, Germany; 2 Department of Leukemia, The University of Texas MD Anderson Cancer Center, Houston, TX USA

**Keywords:** Dasatinib, Chronic myeloid leukemia, First-line treatment, Second-line treatment, Side effects, Early response

## Abstract

**Purpose:**

Dasatinib is a dual Abl/Src tyrosine kinase inhibitor (TKI) designed as a prototypic short-acting BCR–ABL-targeted TKI that inhibits BCR–ABL with greater potency compared with imatinib, nilotinib, bosutinib, and ponatinib and has been shown to have potential immunomodulatory effects. Dasatinib is approved for the treatment of all phases of chronic myeloid leukemia (CML) and Philadelphia chromosome-positive acute lymphoblastic leukemia resistant or intolerant to prior imatinib treatment and first-line treatment for CML in chronic phase. In this article, the development of dasatinib as a treatment for patients with CML is reviewed.

**Methods:**

This is a review of the relevant literature regarding dasatinib development in CML (2003–2013).

**Results:**

Dasatinib demonstrates efficacy against most BCR–ABL mutations arising during imatinib therapy and is effective in treating patients with imatinib resistance due to other mechanisms. Randomized trial data show that first-line dasatinib provides superior responses compared with imatinib and enables patients to achieve early, deep responses correlated with improved longer-term outcomes. Dasatinib has a generally acceptable safety profile, with most adverse events (AEs) proving manageable and reversible. Cytopenias are commonly observed with dasatinib, and some nonhematologic AEs including pleural effusion have been consistently reported.

**Conclusion:**

Dasatinib is an effective treatment option for patients with CML.

## Introduction

Chronic myeloid leukemia (CML) is a malignant clonal disorder of hematopoietic stem cells caused by a chromosomal aberration, the Philadelphia (Ph) chromosome, formed by the chromosomal translocation t(9;22)(q34; q11). This translocation juxtaposes the ABL gene (chromosome 9) and the BCR gene (chromosome 22), creating a BCR–ABL fusion gene. The resulting chimeric protein is a constitutively active ABL tyrosine kinase (Hehlmann et al. 2007). Knowledge of the molecular pathogenesis of CML has allowed the development of molecular-targeted therapy, which has considerably changed the management and outcome of patients (Hehlmann et al. 2007; Wong and Witte 2004). Treatment options for CML include BCR–ABL tyrosine kinase inhibitors (TKIs), interferon alpha, chemotherapy, stem cell transplantation, or clinical trials of novel therapies (Baccarani et al. 2013; NCCN v4. 2013). 

Imatinib was the first BCR–ABL inhibitor developed for CML. Although effective, imatinib is associated with resistance and/or intolerance that reduce its effectiveness in a subset of patients who require alternative treatment options. With first-line imatinib 400 mg once-daily treatment of CML in chronic phase (CML-CP), 65–72 % of patients achieve a complete cytogenetic response (CCyR) and 22–57 % achieve a major molecular response (MMR) by 12 months (Druker et al. 2006; Hochhaus et al. 2009a; Hughes et al. 2003; Kantarjian et al. 2010; Saglio et al. 2010a).

Newer BCR–ABL inhibitors (dasatinib, nilotinib, bosutinib, and ponatinib) were developed to overcome imatinib resistance/intolerance, and most are approved for the second-line treatment for chronic phase (CP), accelerated phase (AP), or blast phase (BP) CML or Ph+ acute lymphoblastic leukemia (ALL; dasatinib and ponatinib only) resistant or intolerant to prior imatinib treatment (nilotinib is not approved for CML-BP). Dasatinib 100 mg once daily and nilotinib 300 mg twice daily are also approved as first-line treatment in CML-CP based on superior efficacy versus imatinib in newly diagnosed patients (EMA Sprycel^®^ [dasatinib] 2012; Kantarjian et al. 2010; Saglio et al. 2010a; Sprycel^®^ BMS 2013; Tasigna^®^ Novartis 2013).

Nilotinib and imatinib have similar chemical structures, with nilotinib showing an improved topographical fit in the BCR–ABL kinase pocket. Whereas imatinib, nilotinib, and ponatinib appear to bind only to the inactive conformation of the kinase, dasatinib is structurally different and binds to both the inactive and active conformations; bosutinib binds to the inactive and intermediate state of the protein, and potentially to the active conformation (Cortes et al. 2010a; Levinson and Boxer 2012; O’Hare et al. 2005, 2009; Puttini et al. 2006; Redaelli et al. 2009; Tokarski et al. 2006; Vajpai et al. 2008; Weisberg et al. 2005; Zhou et al. 2011).

Dasatinib is a potent multikinase inhibitor targeting BCR–ABL, the SRC family of kinases (SRC, LCK, HCK, YES, FYN, FGR, BLK, LYN, FRK), receptor tyrosine kinases (c-KIT, PDGFR, DDR1 and 2, c-FMS, ephrin receptors), and TEC family kinases (TEC and BTK) and demonstrates activity against most imatinib-resistant BCR–ABL mutations (Branford et al. 2009; Karaman et al. 2008; Shah et al. 2004). Although immunosuppressive effects were initially observed in preclinical studies of dasatinib, recent evidence suggests dasatinib may activate and mobilize anti-leukemic immune responses, which may improve efficacy. These immunomodulatory effects may also be implicated in the clinically relevant side effects observed with dasatinib treatment (Das et al. 2005; Kreutzman et al. 2010, 2011; Mustjoki et al. 2010, 2011, 2013; Rix et al. 2007). The recommended dose of dasatinib is 100 mg once daily for CML-CP and 140 mg for CML-AP, CML-BP, or Ph+ ALL administered orally, with or without a meal, because effects of food on dasatinib pharmacokinetics were not clinically relevant in a study of 54 healthy subjects (EMA Sprycel^®^ [dasatinib] 2012; Sprycel^®^ BMS 2013). Following oral administration, maximum plasma concentrations of dasatinib are observed between 0.5 and 6 h. Over the dose range of 15–240 mg/day, dasatinib exhibits dose-proportional increases in the area under the curve (AUC) and linear elimination characteristics. Overall, mean terminal half-life of dasatinib is 3–5 h (Sprycel^®^ BMS 2013; Wang et al. 2013).

As the number of first-line treatments for newly diagnosed CML-CP continues to expand, it is important to understand the profile of each therapy in order to select the most appropriate option for each patient. The effect of dasatinib’s activity profile (high potency, broad spectrum kinase inhibition, potential immune activity) on efficacy and side effect profile in patients with CML-CP will be reviewed in this article.

## In vitro development of dasatinib

Dasatinib was discovered by and named after Jagabandhu Das (Das et al. 2006) as part of an effort to develop potent inhibitors of Src family kinases (SFKs). Kinase selectivity panel screening of dasatinib’s parent compound demonstrated its potency against BCR–ABL and other kinases. Dasatinib was selected for further development based on its activity in a xenograft model of CML and favorable pharmacokinetic profile following oral dosing (Lombardo et al. 2004). Dasatinib is a prototypic short-acting BCR–ABL-targeted TKI with increased potency (325-fold) compared with imatinib in inhibiting unmutated BCR–ABL (Lombardo et al. 2004; O’Hare et al. 2005).

Imatinib resistance is frequently associated with the acquisition of BCR–ABL point mutations, of which over 100 have been identified (Hochhaus et al. 2011; Quintás-Cardama and Cortes 2009). Dasatinib is active against the majority of clinically relevant imatinib-resistant BCR–ABL mutations, in part due to differing binding constraints, allowing dasatinib to bind more effectively to certain imatinib-resistant BCR–ABL mutants (Hochhaus et al. 2011; Tokarski et al. 2006). Although dasatinib, like imatinib, binds to the ATP-binding pocket of BCR–ABL, its binding site only partially overlaps with imatinib-binding sites. Crystal structures of the inhibitors bound to ABL show dasatinib has fewer interactions with the P-loop and interacts less with the activation loop and α-helix compared with imatinib (Tokarski et al. 2006). Based on in vitro assays, dasatinib demonstrates little or no activity against T315I and V299L (IC_50_ > 15 nM); low activity (IC_50_ 5–15 nM) against Y253F, E255 K/V, F317L; and, depending on the study, low activity against G250E, Q252H, and Y253H (Burgess et al. 2005; O’Hare et al. 2005; Redaelli et al. 2009). Patients treated with second-line dasatinib after developing a BCR–ABL mutation on imatinib had markedly reduced response rates (compared to patients with no mutations) if they had T315I or F317L, and possibly lower response rates if carrying Q252H, E255 K, or E355G (Apperley et al. 2009; Cortes et al. 2007b; Guilhot et al. 2007; Hochhaus et al. 2007; Müller et al. 2009; Soverini et al. 2006, Talpaz et al. 2006). Mutations arising during dasatinib treatment include T315I/A, F317L/I/C/V, V299L, and G250E (Cortes et al. 2007b; Hochhaus et al. 2012a; Khorashad et al. 2008; Müller et al. 2009; Shah et al. 2007; Soverini et al. 2007a, b, 2009).

Other kinases potently inhibited by dasatinib include SRC family kinases (SRC, LCK, LYN, YES, FYN, FRK), receptor tyrosine kinases (KIT, EPHA2, and PDGFRα and β), and TEC family kinases (TEC and BTK) (Chang et al. 2008; Dewaele et al. 2010; Hantschel et al. 2007; Huang et al. 2007; Li et al. 2010; Lombardo et al. 2004). The multikinase activity of dasatinib may have therapeutic advantages. Pathologic SFK activity may contribute to BCR–ABL-independent imatinib resistance in CML (Donato et al. 2003; Pene-Dumitrescu and Smithall 2010). Another mechanism of BCR–ABL-independent imatinib resistance is mediated by altered expression of drug influx and efflux proteins, including OCT-1 (White et al. 2007). As dasatinib is not a substrate of OCT-1, its activity is unlikely to be affected by OCT-1 overexpression, in contrast to imatinib (Hiwase et al. 2008).

## Clinical investigations of dasatinib

### The evolution of optimal dasatinib dosing: maintaining clinical efficacy with reduced toxicity

The efficacy of oral dasatinib was first assessed in a phase I, open-label, dose-escalation study (Table 1). Patients (*n* = 84) with various phases of CML or Ph+ ALL intolerant or resistant to imatinib received oral dasatinib (15–240 mg/day) once or twice daily in 4-week treatment cycles (Talpaz et al. 2006). Dasatinib had clinical activity in all CML phases and Ph+ ALL. Complete hematologic response (CHR) was achieved in 92 % of patients (37/40) with CML-CP, and major hematologic response (MHR) was seen in 70 % of patients (31/44) with CML-AP, CML-BP, or Ph+ ALL. The rates of major cytogenetic response (MCyR) were 45 % (18/40) in patients with CML-CP and 43 % (19/44) in patients with CML-AP, CML-BP, or Ph+ ALL. Of note, imatinib-associated side effects, including muscle cramps and nausea, were infrequent with dasatinib, and patients intolerant to imatinib did not have recurrence of the same nonhematologic adverse events (AEs) (e.g., rash and liver function abnormalities) with dasatinib treatment. The major AE associated with dasatinib was reversible myelosuppression. Because dasatinib has a relatively short half-life (3–5 h) (Sprycel^®^ BMS 2013; Wang et al. 2013), the probability of achieving more continuous BCR–ABL inhibition was thought to be increased by twice-daily dosing (Shah et al. 2010); however, once-daily regimens of dasatinib had similar rates of hematologic and cytogenetic response compared with twice-daily regimens and lower rates of AEs supported by a recent retrospective dasatinib exposure–response analysis (Saglio et al. 2010b; Shah et al. 2008a, 2010; Wang et al. 2013).

**Table 1:** Efficacy data from the phase I dasatinib dose-escalation study and phase II START clinical program of second-line dasatinib in patients with different phases of CML resistant and/or intolerant to imatinib therapy (Apperley et al. 2009; Kantarjian et al. 2009a; Mauro et al. 2008; Saglio et al. 2008; Talpaz et al. 2006)

Study/phase	Population	Follow-up (months)	Dose schedule	*N*	Patients, %					
					CHR	MCyR	CCyR	MMR	PFS	OS
CA180-002 phase I	CML-CP, CML-AP, CML-BP, or Ph+ ALL imatinib R/I	Median 12	15 to 240 mg dasatinib per day	40 (CP)	92	45	35	−	−	−
				11 (AP)	45	27	18	−	−	−
				23 (MBP)	35	35	26	−	−	−
				10 (LBP, Ph+ ALL)	70	80	30	−	−	−
START-A phase II	CML-AP imatinib R/I	Median 14.1	70 mg dasatinib twice daily	174	45	39	32	−	66	82
START-B phase II	CML-MBP imatinib R/I	Minimum 24	70 mg dasatinib twice daily	109	26	34	27	−	−	38
START-L phase II	CML-LBP^a^ imatinib R/I	Minimum 24	70 mg dasatinib twice daily	48	29	52	46	−	−	26
START-C phase II	CML-CP imatinib R/I	Minimum 24	70 mg dasatinib twice daily	387	91	62	53	47	80	94
START-R phase II	CML-CP imatinib R	Minimum 24	70 mg dasatinib twice daily	101	93	53	44	29	86	−
			400 mg imatinib twice daily	49	82	33	18	12	65	−

*AP* accelerated phase, *BP* blast phase, *CCyR* complete cytogenetic response, *CHR* complete hematologic response, *CP* chronic phase, *I* intolerant, *L* lymphoid, *M* myeloid, *MCyR* major cytogenetic response, *MMR* major molecular response, *Ph+ ALL* Philadelphia chromosome-positive acute lymphoblastic leukemia, *OS* overall survival, *PFS* progression-free survival, *R* resistant

^a^START-L also included a Ph+ ALL cohort, data not reported here

A series of phase II trials, the pivotal START (SRC–ABL Tyrosine kinase inhibition Activity Research Trials) trial program (Table 1), followed the phase I dose-escalation study. The primary objective for these trials was to treat patients with resistance or intolerance to imatinib who therefore had a life-threatening medical need. As the pharmacokinetics of the dasatinib 70 mg twice-daily regimen were better understood, it was selected as the preferred dosing option in these patients. These open-label, multicenter trials established the efficacy and safety of second-line dasatinib (70 mg twice daily) in the treatment of imatinib-resistant or imatinib-intolerant patients with CML (all phases) or Ph+ ALL. Data from this program led to the initial approval of dasatinib in these indications.

Two START studies assessed second-line dasatinib 70 mg twice daily in patients with CML-CP. START-C was a single-arm study, and START-R was a randomized, parallel-arm study of dasatinib versus high-dose imatinib (800 mg/day) in patients resistant to standard dose imatinib (Hochhaus et al. 2007, 2008; Kantarjian et al. 2007, 2009a; Mauro et al. 2008). In START-C (*N* = 387), dasatinib induced MCyR (primary end point) in 62 % of patients after a minimum follow-up of 24 months (Mauro et al. 2008). The corresponding CCyR rate was 53 %. In START-R, rates of MCyR were 53 % in the dasatinib 70 mg twice-daily arm (*n* = 101) and 33 % in the high-dose imatinib arm (*n* = 49) (*p* = 0.017) after a minimum follow-up of 24 months (Kantarjian et al. 2009a). CCyR rates were 44 and 18 %, respectively (*p* = 0.0025). Although no formal statistical comparison between the study arms was planned, the data suggested relatively greater efficacy for dasatinib compared with imatinib (Kantarjian et al. 2009a). These responses were also durable, as a pooled analysis (*N* = 387) of the START-C and START-R studies showed that 90 % of patients achieving a CCyR maintained this level of response after 24 months (Baccarani et al. 2008). START-A, START-B, and START-L were single-arm studies of second-line dasatinib 70 mg twice daily in patients with CML-AP, CML-BP, and CML-BP/Ph+ ALL, respectively (Apperley et al. 2009; Cortes et al. 2007a, 2008; Guilhot et al. 2007; Ottmann et al. 2007; Saglio et al. 2008). In the START-A trial (*N* = 174), after a median follow-up of 14.1 months, 64 % of patients with CML-AP achieved the primary end point of MHR and 45 % achieved a CHR (Apperley et al. 2009). START-B included patients with myeloid CML-BP (*N* = 109), and START-L included patients with lymphoid CML-BP (*n* = 48) and a subset of patients with Ph+ ALL (Cortes et al. 2007a). After a minimum follow-up of 24 months, a CHR was achieved in 26 % of patients with myeloid CML-BP and in 29 % of patients with lymphoid CML-BP (Saglio et al. 2008).

The recommended starting dose for dasatinib in patients with CML-CP is now 100 mg once daily (EMA Sprycel^®^ [dasatinib] 2012; Sprycel^®^ BMS 2013) following the results of a phase III dose-optimization study showing that 100 mg once daily was associated with similar efficacy as the twice-daily regimen, but with a reduction in toxicity (Shah et al. 2008a). Although the dasatinib half-life of 3–5 h (Sprycel^®^ BMS 2013) was used as a basis for the initial twice-daily dosing regimen, transient exposure of CML cell lines to dasatinib in vitro has been demonstrated to induce apoptosis (Shah et al. 2008b), supporting the feasibility of once-daily dosing, and data from the phase I study suggested that once-daily and twice-daily dose schedules were associated with similar response rates (Talpaz et al. 2006). Furthermore, due to dose reductions in the START-C and START-R studies, the median total daily dose delivered to patients approximated 100 mg/day (Hochhaus et al. 2007; Kantarjian et al. 2007). It was therefore proposed to compare the 100 mg once-daily dose with other schedules. In this dose-optimization study, patients (*N* = 670) were randomized to receive dasatinib at 100 mg once daily (*n* = 167), 140 mg once daily (*n* = 167), 50 mg twice daily (*n* = 168), or 70 mg twice daily (*n* = 168) (Shah et al. 2008a). After a minimum follow-up of 2 years, rates of CCyR and MMR were similar across the different dosing schedules (CCyR 50–54 %; MMR 37–38 %) (Shah et al. 2010). In the 100 mg once-daily arm, the 24-month rates of CCyR and MMR were 50 and 37 %, respectively. Rates of progression-free survival (PFS), overall survival (OS), and transformation to AP/BP by 24 months were 80, 91, and 3 %, respectively (Table 2). The 100 mg once-daily arm was associated with improved safety. Rates of all-grade pleural effusion (*p* = 0.049), grade ≥3 thrombocytopenia (*p* = 0.003), all-grade neutropenia (*p* = 0.034), and all-grade leukocytopenia (*p* = 0.017) were significantly lower for patients treated with dasatinib 100 mg once daily compared with other schedules (Shah et al. 2010). After a minimum follow-up of 5 years, PFS, OS, and rates of transformation to AP/BP were 57, 78, and 5 %, respectively, in the 100 mg once-daily arm (Shah et al. 2012).

**Table 2:** Efficacy data from the CA180-034 phase III dose-optimization study of second-line dasatinib in patients with CML-CP resistant or intolerant to imatinib therapy after a minimum follow-up of 2 years (Shah et al. 2008a, 2010)

Dasatinib dose schedule	*n*	Patients, %					
		CHR	MCyR	CCyR	MMR	PFS^a^	OS
100 mg once daily	167	92	63	50	37	80	91
70 mg twice daily	168	88	61	54	38	76	88
140 mg once daily	167	87	63	50	38	75	94
50 mg twice daily	168	92	61	50	38	76	90

*CCyR* complete cytogenetic response, *CHR* complete hematologic response, *MCyR* major cytogenetic response, *MMR* major molecular response, *OS* overall survival, *PFS* progression-free survival

^a^Definition of disease progression: loss of previous CHR or MCyR, confirmed AP/BP disease, increasing WBC count (recorded by the investigator as a doubling from the lowest value to >20,000/mm^3^ or increases of >50,000/mm^3^ on 2 assessments ≥2 weeks apart), increase in Ph+ metaphases by ≥30 %, or death from any cause

A similar phase III dose-optimization study in patients with CML-AP (Kantarjian et al. 2009b) and CML-BP (Saglio et al. 2010b) led to a recommended dasatinib dose of 140 mg once daily in these indications (EMA Sprycel^®^ [dasatinib] 2012; Sprycel^®^ BMS 2013). Patients were randomized to receive dasatinib 70 mg twice daily (*n* = 159, AP; *n* = 74, myeloid BP [MBP]; *n* = 28, lymphoid BP [LBP]) or 140 mg once daily (n = 158, AP; *n* = 75 MBP; *n* = 33, LBP). In patients with CML-AP, similar rates of MHR (68 vs 66 %) and MCyR (43 vs 39 %) were observed in both treatment arms after a median follow-up of 15 months. Significantly, fewer patients in the once-daily arm had pleural effusion compared with the twice-daily arm (*p* < 0.001) (Kantarjian et al. 2009b). After 2 years of follow-up, for patients with myeloid BP, the MHR rates in both arms were 28 %; for those with lymphoid BP, the corresponding rates were 42 % in the once-daily arm and 32 % in the twice-daily arm. AE rates were suggestive of improved safety for dasatinib 140 mg once daily (Saglio et al. 2010b).

### Early responses to dasatinib 100 mg once daily in the treatment for newly diagnosed CML-CP

Following the success of second-line dasatinib in treating patients with CML, trials were performed to assess the clinical benefit of this agent in the first-line setting. The rationale for performing first-line studies derives partly from the observation that earlier responses to therapy are associated with better outcomes as seen with first-line imatinib (de Lavallade et al. 2008; Druker et al. 2006; Kantarjian et al. 2008; Marin et al. 2008). Similarly, in a retrospective analysis of patients from the dasatinib START-C and START-R, and dose-optimization trials, patients who achieved CCyR at 12 months had a 24-month PFS rate of 97 % (95 % confidence interval [CI], 93–100 %) compared with 78 % (95 % CI, 72–83 %) for patients who did not achieve CCyR or MMR at this time point (Hochhaus et al. 2009b). In exploratory landmark analyses of the second-line dasatinib dose-optimization study, patients receiving dasatinib 100 mg once daily with cytogenetic assessments at 12 months showing achievement of CCyR had higher PFS after 4-year minimum follow-up compared with patients achieving less than partial CyR (PCyR) responses (87 vs 45 %) (Shah et al. 2011). With 5-year follow-up, similar trends were observed for patients achieving ≤10 % BCR–ABL levels compared to patients with >10 % BCR–ABL levels at 3 months (Shah et al. 2012). These landmark analyses demonstrate the importance of achieving an early response to improve patient outcome. Furthermore, dasatinib is more potent than imatinib, and less susceptible to mechanisms of imatinib resistance. Therefore, it may be expected to elicit earlier responses than imatinib with consequently improved long-term outcomes.

The first trial investigating dasatinib as first-line treatment was a phase II, open-label study (Cortes et al. 2010b). Patients with newly diagnosed CML-CP were randomized to receive dasatinib 100 mg once daily (*n* = 66) or 50 mg twice daily (*n* = 33) (Pemmaraju et al. 2011). Because of results from a phase III multinational randomized study of first-line dasatinib and trends in favor of the 100 mg once-daily schedule of dasatinib seen in this study and others, the 50 mg twice-daily arm of this trial was closed after 66 patients were enrolled, and all subsequent patients were randomized to the 100 mg once-daily arm. The study continues enrolling patients in the once-daily arm (Cortes et al. 2010b; Pemmaraju et al. 2011). After a median follow-up of 29 months, in patients with ≥3-month follow-up (*n* = 87), rates of CCyR and MMR were 95 and 86 %, respectively. BCR–ABL levels of ≤0.0032 % (≥4.5-log reduction; MR^4.5^) were achieved in 67 % of patients. Responses were achieved rapidly with 94 and 95 % of patients achieving a CCyR after 6 and 12 months, respectively. Similarly, MMR rates at 6 and 12 months were 68 and 73 %, respectively. These data compare favorably with historic response data for imatinib (Pemmaraju et al. 2011).

**Fig. 1:**
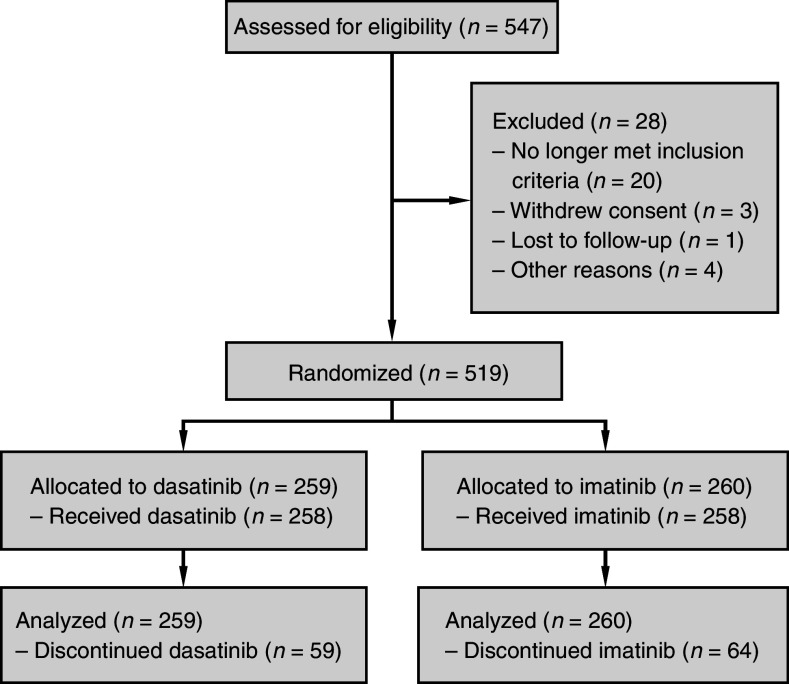
Study design and patient disposition for the DASISION phase III trial of dasatinib versus imatinib in newly diagnosed CML-CP (Kantarjian et al. 2012)

Dasatinib in the first-line setting was further investigated in the pivotal, open-label, multinational, randomized phase III trial of Dasatinib versus Imatinib Study in Treatment-Naïve CML Patients (DASISION) (Kantarjian et al. 2010). In this study, 519 patients newly diagnosed with CML-CP were randomized to receive dasatinib 100 mg once daily (*n* = 259) or imatinib 400 mg once daily (*n* = 260) (Figure 1) (Kantarjian et al. 2010). Efficacy data are shown in Table 3. The primary end point of this study was confirmed CCyR (cCCyR; CCyR on two consecutive assessments) by 12 months. For the dasatinib versus imatinib arms, the rate of cCCyR by 12 months was 77 versus 66 % (*p* = 0.007), respectively (Kantarjian et al. 2010). Cumulative CCyR, MMR, and MR^4.5^ rates were higher for dasatinib across a 24-month period (*p* = 0.0002, *p* < 0.0001, and *p* = 0.002, respectively) (Kantarjian et al. 2012). Responses to dasatinib were rapid and prolonged; median times to CCyR were 3.2 and 6.0 months and median times to MMR were 15 and 36 months in the dasatinib and imatinib arms, respectively (Kantarjian et al. 2012). At 24 months, for dasatinib versus imatinib, cumulative rates of MMR were 64 versus 46 % (*p* < 0.0001), rates for BCR–ABL ≤ 0.01 % (MR^4^) were 29 versus 19 % (*p* = 0.0053), and rates of MR^4.5^ were 17 versus 8 % (*p* = 0.0032) (Hochhaus et al. 2012a; Kantarjian et al. 2012). After 2-year follow-up, transformation to AP/BP throughout study follow-up (including on study and after discontinuation) occurred in nine patients (3.5 %) receiving dasatinib and 15 (5.8 %) receiving imatinib (Hochhaus et al. 2012a; Kantarjian et al. 2012). At 2-year follow-up, survival data for this study remain immature, but no difference was observed between dasatinib and imatinib for PFS (93.7 and 92.1 %) and OS (95.3 and 95.2 %). A small difference in failure-free survival for dasatinib versus imatinib was observed (including protocol defined progression; 91.2 vs 87.8 %) (Hochhaus et al. 2012a; Kantarjian et al. 2012).

**Table 3:** Efficacy data from the DASISION phase III trial of first-line dasatinib versus first-line imatinib in patients with newly diagnosed CML-CP after a minimum follow-up of 2 years (Kantarjian et al. 2012)

Treatment arm	*n*	Patients, %							
		CCyR	cCCyR	MMR	MR^4^	MR^4.5^	PFS^a^	FFS^b^	OS
Dasatinib 100 mg once daily	259	86	80	64	29	17	94	91	95
Imatinib 400 mg once daily	260	82	74	46	19	8	92	88	95

*AP* accelerated phase, *cCCyR* confirmed CCyR (CCyR on two separate assessments 28 days apart), *CCyR* complete cytogenetic response, *CHR* complete hematologic response, *CP* chronic phase, *FFS* failure-free survival, *MCyR* major cytogenetic response, *MMR* major molecular response, *MR* ^*4*^ BCR–ABL ≤0.01 % (≥4-log reduction in BCR–ABL levels), *MR* ^*4.5*^ BCR–ABL ≤0.0032 %  (≥4.5-log reduction in BCR–ABL levels), *OS* overall survival, *PCyR* partial cytogenetic response, *PFS* progression-free survival

^a^Definition of disease progression: development of CML-AP/BP, doubling of white blood cell count to >20 × 10^9^/L in the absence of CHR, loss of CHR, increase in Ph+ bone marrow metaphases to >35 %, death from any cause

^b^Definition of failure: no hematologic response by 3 months, no CHR or cytogenetic response by 6 months, no PCyR by 12 months, or no CCyR by 18 months, or progression as defined above

In exploratory analyses, achieving an early molecular response (BCR–ABL levels of ≤10 %) at 3 months was associated with lower transformation rates (dasatinib: 1.5 vs 8.1 %; imatinib: 2.6 vs 9.4 %), better long-term outcomes (24-month PFS: dasatinib, 97 vs 83 %; imatinib, 96 vs 85 %), and improved response (24-month MMR rates: dasatinib, 76 vs 16 %; imatinib, 66 vs 19 %) in both treatment arms (Hochhaus et al. 2012b). Deeper levels of response were achieved earlier with dasatinib compared with imatinib as equivalent BCR–ABL (international scale [IS]) levels were achieved 6 months earlier with dasatinib, and a higher proportion of patients receiving dasatinib achieved BCR–ABL levels of ≤10 % at 3 months compared with patients receiving imatinib (84 vs 64 %) (Hochhaus et al. 2012b; Saglio et al. 2012). Similar results were found in another first-line study of dasatinib. Results from exploratory analyses of the dasatinib arm of the SPIRIT 2 trial have been reported, and after 2 years of follow-up, 91.4 % of patients receiving dasatinib achieved BCR–ABL levels of ≤10 % at 3 months (Marin et al. 2012a). Compared with patients who had >10 % BCR–ABL levels, patients achieving ≤10 % BCR–ABL at 3 months had significantly higher 2-year cumulative rates of CCyR (91.4 vs 58.8 %, *p* < 0.001), MMR (79.8 vs 14.3 %, *p* < 0.001), and MR^4.5^ (45.7 vs 0 %, *p* < 0.001) (Marin et al. 2012a).

In total, 23 % of dasatinib-treated patients and 25 % of imatinib-treated patients discontinued treatment in DASISION; 5 and 7 % due to study-defined disease progression (defined as any of the following: doubling of white cell count to >20 × 10^9^/L in the absence of CHR; loss of CHR; increase in Ph-positive metaphases to >35 %; transformation to AP/BP; death from any cause), 3 and 4 % due to treatment failure, and 7 and 5 % due to drug-related AEs, respectively (Kantarjian et al. 2012). In patients who discontinued treatment, BCR–ABL mutations were found in 10 patients in each arm, with a narrower spectrum of mutations seen with dasatinib versus imatinib (3 vs 9 different amino acids affected). Mutations associated with discontinuation in the dasatinib arm were T315I (*n* = 7), F317L (*n* = 2), and F317I/V299L (*n* = 1) (Kantarjian et al. 2012).

Similar levels of response have been observed in additional studies of first-line dasatinib. In the SWOG S0325 phase II study, newly diagnosed patients were randomized to receive dasatinib 100 mg once daily (*n* = 123) or imatinib 400 mg once daily (*n* = 123) (Radich et al. 2012). At 12 months, median reductions in BCR–ABL transcript levels were greater with dasatinib compared with imatinib (3.3 vs 2.8 log; *p* = 0.063), as were the rates of >3-log BCR–ABL reductions (59 vs 44 %; *p* = 0.059). Rate of CCyR was significantly different between the dasatinib and imatinib arms (84 and 69 %, respectively; *p* = 0.040), although cytogenetic responses were only assessed in 53 % of patients (Radich et al. 2012).

### Side effects or adverse events

Since early clinical trials, some AEs have been consistently reported in patients receiving dasatinib, including myelosuppression, fluid retention, pleural effusion, gastrointestinal disorders, fatigue, headache, musculoskeletal disorders, rash, and infection. Some bleeding events have also been reported. More recently, cases of pulmonary arterial hypertension (PAH), a sub-category of pulmonary hypertension (PH) and atypical of classical PAH with at least partial reversibility upon drug discontinuation, have been reported in a small number of patients receiving dasatinib (Dumitrescu et al. 2011; Fang et al. 2012; Galiè et al. 2009; Hennigs et al. 2011; Mattei et al. 2009; McLaughlin et al. 2009; Montani et al. 2012; Orlandi et al. 2011; Philibert et al. 2011; Rasheed et al. 2009; Sano et al. 2012). In clinical trials of first-line and second-line dasatinib, most AEs occurred within 12–24 months of treatment and were managed with dose modifications (Kantarjian et al. 2012; Shah et al. 2012; Sprycel^®^ BMS 2013).

In the early phase I, open-label, dose-escalation study, the major AE was reversible myelosuppression, leading to dose interruption in 60 % of patients (Talpaz et al. 2006). Grade 3/4 neutropenia and thrombocytopenia were seen in 45 and 35 % of patients with CML-CP, respectively. Nonhematologic AEs included diarrhea, nausea, and peripheral edema. Treatment-related pleural effusion occurred in 13 % of patients with CML-CP (Talpaz et al. 2006). Rates of AEs in this study may be expected to be elevated, as some patients received doses of dasatinib considerably higher than the current recommended dose of 100 mg once daily (range of dasatinib dose received 15–240 mg/day). A maximum tolerated dose was not determined in this study, and no patient withdrew from treatment as a result of toxic effects (Talpaz et al. 2006).

In the following START-C phase II trial, in which patients with CML-CP received second-line dasatinib 70 mg twice daily, 9 % of patients discontinued treatment because of study drug toxicity after 8 months of follow-up (Hochhaus et al. 2007). Cytopenias were common (grade 3/4 thrombocytopenia, 47 %; neutropenia, 49 %), but generally reversible and manageable with dose adjustments. Pleural effusion was observed in 19 % of patients (grade 3/4 in 3 %) (Hochhaus et al. 2007). Similar results were seen in the START-R phase II trial of dasatinib 70 mg twice daily (Kantarjian et al. 2007). After a median follow-up of 15 months, 28 % of patients had discontinued treatment, 16 % due to study drug intolerance. Cytopenias were common (grade 3/4 thrombocytopenia, 56 %; neutropenia, 61 %) but reversible, and manageable with dose modification. Pleural effusion occurred in 17 % of patients (Kantarjian et al. 2007). Most cases of pleural effusion observed across the START studies were managed with temporary dose interruption, diuretics, or pulse steroid therapy (Apperley et al. 2009; Cortes et al. 2007a; Hochhaus et al. 2007; Kantarjian et al. 2007). In the START-C and START-R trials, patients received dasatinib at 70 mg twice daily, which is higher than the current recommended dose for CML-CP (100 mg once daily). It may therefore be expected that the frequency of AEs and the rate of discontinuation due to study drug intolerance might be higher than expected in these trials compared with patients receiving the current recommended dose for CML-CP.

A single institution subgroup analysis of 138 patients treated with dasatinib in the phase I dose-escalation study and phase II START trials showed that 29 % of patients with CML-CP developed pleural effusion (Quintás-Cardama et al. 2007). Patients receiving 100 mg once-daily dasatinib had a lower incidence of pleural effusion compared with patients receiving 50 or 70 mg twice daily, or 140 mg once daily, while efficacy remained consistent across all four dosing schedules. Furthermore, a separate analysis indicated that intermittent dosing of dasatinib at 100 mg per day for 5 days per week, including a weekend drug holiday where dasatinib was not taken, led to reductions in the rate and severity of AEs including fluid retention and pleural effusion, while efficacy and disease control were maintained (La Rosée et al. 2013). An analysis of risk factors for pleural effusion in patients treated with second-line dasatinib identified prior history of cardiac disease (*p* = 0.02), hypertension (*p* = 0.01), and twice-daily dosing schedule (*p* = 0.05) to be associated with an increased risk of pleural effusion (Quintás-Cardama et al. 2007). In a separate analysis, older age was the only baseline characteristic associated with an increased risk of pleural effusion (Porkka et al. 2010). The development of lymphocytosis during dasatinib treatment was associated with a 1.7-fold increased risk of pleural effusion (95 % CI, 1.1–2.5) (Porkka et al. 2010).

The second-line, phase III dose-optimization study indicated that dasatinib 100 mg once daily was associated with reduced frequency of AEs compared with twice-daily dosing regimens in patients with CML-CP, while efficacy was maintained (Porkka et al. 2010; Shah et al. 2008a, 2012). With a minimum follow-up of 6 months, patients receiving dasatinib 100 mg once daily had lower rates of pleural effusion and grade 3/4 thrombocytopenia compared with patients receiving 70 mg twice daily (7 vs 16 % and 22 vs 37 %, respectively) (Shah et al. 2008a). Fewer patients receiving dasatinib 100 mg once daily required dose interruptions (51 vs 68 %), dose reductions (30 vs 55 %), or discontinuation (16 vs 23 %) (Shah et al. 2008a). With a minimum follow-up of 24 months, 14 % of patients receiving dasatinib 100 mg once daily developed pleural effusion, compared with 25 % of patients receiving 70 mg twice daily (Porkka et al. 2010). Improved tolerability of once-daily dosing may be due to intermittent dasatinib exposure in comparison with continuous exposure achieved by twice-daily dosing (Porkka et al. 2010). After a minimum follow-up of 5 years, grade 3/4 hematologic AEs in the 100 mg once-daily arm included neutropenia (36 %) and thrombocytopenia (24 %). Any-grade nonhematologic AEs included headache (33 %), diarrhea (28 %), fatigue (26 %), and pleural effusion (24 %) (Shah et al. 2012). Grade 3/4 cytopenias and any-grade nonhematologic AEs generally first occurred within 12–24 months of treatment (Shah et al. 2012).

In the first-line setting, similar AEs were observed. Treatment-related AEs led to the discontinuation of dasatinib in 7 % of patients (Kantarjian et al. 2012). Grade 3/4 hematologic AEs were relatively common in patients with CML-CP receiving dasatinib (100 mg once daily) or imatinib (400 mg once daily) in DASISION, after a minimum follow-up of 24 months (neutropenia: 24 vs 21 %; thrombocytopenia: 19 vs 11 %; anemia: 11 vs 8 %) (Kantarjian et al. 2012). Severe biochemical abnormalities were uncommon with the exception of grade 3/4 hypophosphatemia (dasatinib arm, 7 %; imatinib arm, 25 %) (Kantarjian et al. 2012). The most common nonhematologic AEs in DASISION (all grades, dasatinib vs imatinib) were myalgia (22 vs 39 %), diarrhea (19 vs 21 %), pleural effusion (14 vs 0 %), headache (13 vs 11 %), superficial edema (11 vs 36 %), rash (11 vs 17 %), and nausea (10 vs 23 %) (Kantarjian et al. 2012). Grade 3/4 nonhematologic AEs associated with dasatinib were uncommon at 0–2 % (fluid retention, 2 %; pleural effusion, 1 %; diarrhea, <1 %; fatigue, <1 %) (Kantarjian et al. 2012). In DASISION, at 1-year follow-up, 26 patients (10 %) had pleural effusion; all events were grade 1 (2 %) or grade 2 (8 %) (Kantarjian et al. 2010). By 2-year follow-up, pleural effusion events had occurred in 37 patients (14.3 %) and were generally mild-to-moderate in severity (grade 1: *n* = 9, 3.5 %; grade 2: *n* = 26, 10.1 %; grade 3: *n* = 2, 0.8 %) with no grade 4 events observed. Events were largely manageable with treatment interruption (*n* = 30), dose reduction (*n* = 19), or the use of diuretics (*n* = 17) or corticosteroids (*n* = 15). Four patients required a therapeutic thoracentesis. At 2-year follow-up, five patients (1.9 %) had discontinued dasatinib due to pleural effusion. Notably, the occurrence and management of pleural effusion appeared not to affect the efficacy of dasatinib (Kantarjian et al. 2012; Laneuville et al. 2011).

In some patients receiving dasatinib, large granular lymphocyte (LGL) expansions carrying clonal T-cell receptor gene arrangements occur resulting in lymphocytosis (Kreutzman et al. 2010). Data from a retrospective analysis of patients enrolled in DASISION suggested that dasatinib-treated patients with lymphocytosis had higher rates of any-grade pleural effusion and lower rates of myalgias and arthralgias compared with patients without lymphocytosis (Schiffer et al. 2010a). In a separate analysis of pooled study data, 31 % of patients with CML-CP had lymphocytosis, which was associated with a higher rate of CCyR and longer PFS in patients with advanced disease (Schiffer et al. 2010b). However, no formal statistical testing has been reported for either of these analyses. A subanalysis of DASISION demonstrated no substantial effects of baseline cardiovascular conditions, other comorbidities, or use of baseline medications on the side effects of dasatinib (Guilhot et al. 2010; Khoury et al. 2010; Saglio et al. 2010c).

More recently, rare cases of PAH in patients receiving dasatinib for CML and Ph+ ALL have been reported in the literature (*n* = 16) (Dumitrescu et al. 2011; Hennigs et al. 2011; Mattei et al. 2009; Montani et al. 2012; Orlandi et al. 2011; Philibert et al. 2011; Rasheed et al. 2009; Sano et al. 2012). By 2-year follow-up of the phase III DASISION trial of dasatinib versus imatinib in newly diagnosed CML-CP, three patients receiving dasatinib developed PH; however, no cases of PAH diagnosed by right heart catheterization (RHC) were recorded (Kantarjian et al. 2012). No patient in DASISION discontinued dasatinib therapy because of PH or PAH (Kantarjian et al. 2012). PAH observed in patients receiving dasatinib is not typical, as this disease is normally progressive, including cases with a drug-induced etiology which do not reverse on treatment withdrawal (Galiè et al. 2009; McLaughlin et al. 2009). To date, however, the typical clinical course for dasatinib-associated cases of PAH is improvement or complete resolution in the majority of cases upon withdrawal of treatment.

Guidelines for the management of AEs occurring in patients receiving dasatinib treatment are largely based on the experience of clinicians treating patients in early clinical trials (Khoury et al. 2009; Quintás-Cardama et al. 2008). For most AEs occurring in patients receiving dasatinib treatment, guidelines recommend dose interruption or dose reduction (NCCN v4. 2013; Sprycel^®^ BMS 2013). Early studies showed that cytopenias were usually reversible and effectively managed with dose interruption or reduction, with a minority of cases requiring blood transfusions or hospitalization (Apperley et al. 2009; Brave et al. 2008; Cortes et al. 2007a, 2008; Guilhot et al. 2007; Hochhaus et al. 2007; Ottmann et al. 2007; Quintás-Cardama et al. 2009b; Serpa et al. 2010; Shah et al. 2008a). In one study, cytopenias resolved in 60 % of patients upon interruption (Talpaz et al. 2006); in another study, permanent discontinuation was required in only 1 % (Brave et al. 2008). If hematologic AEs occur in patients receiving dasatinib, treatment should be interrupted until the absolute neutrophil count is ≥1.0 × 10^9^/L and platelets ≥50 × 10^9^/L. Dasatinib can then be resumed at the original dose if recovery occurs within 7 days or at a reduced dose of 80/50 mg/day if recovery takes longer than 7 days or if the event was a second/third recurrence. Although not yet licensed in all regions, growth factor support may also be useful for managing hematologic AEs (NCCN v4. 2013; Quintás-Cardama et al. 2009b; Shah et al. 2008a; Sprycel^®^ BMS 2013). If a severe nonhematologic AE (grade 3/4) develops, guidelines indicate that dasatinib be withheld until resolution or improvement. Treatment can then be resumed at a reduced dose dependent on initial severity of the event (NCCN v4. 2013; Sprycel^®^ BMS 2013). Early reports indicate that most nonhematologic AEs, including neuropathy, dyspnea, elevated liver enzymes, headache, bone pain, rash, renal failure, cardiac abnormality, infections, pancreatitis, and diarrhea, were effectively managed with dose reductions or interruptions (Apperley et al. 2009; Cortes et al. 2008; Hochhaus et al. 2007; Serpa et al. 2010). Consistent with reports, guidelines indicate that most pleural effusion events can be managed through dose reduction or interruption, and/or corticosteroids and diuretics, with a minority of cases requiring thoracentesis, oxygen therapy, or pleurodesis (Brave et al. 2008; Cortes et al. 2007a, 2008; Guilhot et al. 2007; Hochhaus et al. 2007; Kantarjian et al. 2012; Laneuville et al. 2011; Shah et al. 2008a; Talpaz et al. 2006). Once resolved, treatment can be resumed at the same or at a reduced dasatinib dose depending on event severity. A retrospective analysis of an intermittent treatment schedule of dasatinib at different doses demonstrated a reduction in the grade of pleural effusion and in hematologic toxicity without compromising efficacy (La Rosée et al. 2013). Other fluid retention events can be managed with diuretics and supportive care. To reduce the risk of PAH, patients should be evaluated for signs and symptoms of underlying cardiopulmonary disease before initiating dasatinib treatment. Upon confirmation of a PAH diagnosis based on RHC, guidelines indicate that dasatinib should be permanently discontinued (NCCN v4. 2013; Sprycel^®^ BMS 2013). PAH may be at least partially reversible upon treatment discontinuation. For bleeding events, recommended management steps include dose interruption and transfusion (Quintás-Cardama et al. 2009a; Sprycel^®^ BMS 2013). Rash may be managed with topical or systemic steroids, in addition to dose reduction, interruption, or discontinuation. Specific supportive medication is also indicated in case of headache and diarrhea (NCCN v4. 2013; Sprycel^®^ BMS 2013). A subanalysis of DASISION showed that dose modifications taken to manage AEs had no apparent effect on response (Jabbour et al. 2011).

## Conclusions

Dasatinib has superior efficacy over imatinib and manageable side effects in first-line and second-line treatment of patients with CML. The potent, multi-targeted activity of dasatinib may contribute to the depth and speed of response achieved with this agent. Dasatinib’s potential immune activity may play a role in the observed potency and requires further investigation. These factors may also play a role in the safety profile and the AEs observed in patients receiving dasatinib.

In exploratory analyses, a greater proportion of patients achieved early, deep molecular responses (≤10 % BCR–ABL at 3 months) with dasatinib compared with imatinib. Earlier, deeper responses with either TKI were associated with improved response and survival and decreased transformation to AP/BP. With significantly deeper levels of molecular response achieved at all time points with up to 2-year follow-up in DASISION, more patients receiving dasatinib versus imatinib may achieve undetectable levels of BCR–ABL transcripts and a complete molecular response. Second-generation BCR–ABL inhibitors have also demonstrated some activity against CML stem cells, providing support for future investigation into dasatinib in achieving a molecular cure (Defina et al. 2012; Hiwase et al. 2010; Mustjoki et al. 2011). A phase II study is currently investigating whether CML-CP patients with a sustained complete molecular response (12 months; ≤0.0032 % or 4.5-log reduction of BCR–ABL transcript from standardized baseline) on dasatinib maintain undetectable or minimally detectable BCR–ABL residual disease upon treatment discontinuation (BMS 2013a).

With changing treatment goals supporting earlier, deeper responses, it is reasonable to suggest that second-generation BCR–ABL inhibitors are likely to be used more frequently as a first-line treatment option in patients with newly diagnosed disease, dependent on existing patient comorbidities and BCR–ABL mutation status (if known). The speed of response achieved with second-generation BCR–ABL inhibitors may also allow the early identification of a subset of patients resistant to BCR–ABL inhibitor treatment who may benefit from alternate therapy (stem cell transplant or clinical trials).

The loss of patent exclusivity for imatinib in 2015 (USA) and 2016 (EU) may influence first-line treatment selection. With this potential for increased use of imatinib, it will be important to closely monitor patient response to ensure early milestones are achieved. Data are emerging to evaluate the potential benefit of a change in treatment for patients failing to reach certain levels of response (≤10 % BCR–ABL by 3 months) (Hanfstein et al. 2012; Marin et al. 2012a, b). Based on these retrospective analyses, the NCCN guidelines recommend, among several proposed therapies, a change in treatment for patients with >10 % BCR–ABL at 3 months (NCCN v4. 2013). However, there is no data yet showing that such an early change in TKI therapy will improve outcome. A phase II study comparing dasatinib 100 mg once daily to imatinib standard of care in patients failing to achieve an optimal response of ≤10 % BCR–ABL after 3 months of imatinib 400 mg/day is currently in progress (BMS 2013b). This study will test the hypothesis that changing to dasatinib treatment in this patient population will induce an improved response rate (primary end point, MMR at 12 months) compared with continuing imatinib at any dose. Whether this would be associated with differences in long-term outcomes (event-free survival, PFS, OS) remains to be proven.

With the growing number of BCR–ABL inhibitors available for patients with CML-CP and the lack of head-to-head clinical trials across second-generation BCR–ABL inhibitors, choosing a treatment requires consideration on a patient-to-patient basis, and therefore, information regarding the efficacy and use of these agents in the real-world setting is of increasing interest. An observational 5-year prospective cohort study (BMS 2013c) has been initiated to further understand the use of dasatinib, imatinib, and nilotinib in patients with newly diagnosed CML-CP including response, outcomes, treatment adherence, and patient quality of life. Data are anticipated to provide additional information to help guide initial treatment selection.
